# A Standardised Clerking Proforma Improves the Perceived Satisfaction of Healthcare Professionals and Leads to Better Documentation

**DOI:** 10.7759/cureus.98706

**Published:** 2025-12-08

**Authors:** Ahamed T Mohideen, Michael Foxall-Smith, Matthew Arnaouti, Katy Emslie, Devender Mittapalli

**Affiliations:** 1 Vascular Surgery, University Hospitals Plymouth NHS Trust, Plymouth, GBR; 2 Peninsula Medical School, University of Plymouth, Plymouth, GBR

**Keywords:** clerking, documentation, medical record keeping, proforma, surgery

## Abstract

Introduction

High-quality medical records are integral to Good Medical Practice and providing optimal patient care in the United Kingdom. This study was designed to assess the effectiveness of a structured Surgical Assessment Proforma in improving documentation within the surgical assessment unit (SAU) of a major trauma centre.

Methodology

A four-phase prospective study was undertaken using the Plan, Design, Study, and Act (PDSA) methodology. This included an initial clinician survey and proforma development, audit, re-audit post-implementation, and a final user survey. Evaluation and proforma design utilised standards from the Royal College of Surgeons and the Professional Record Standards Body (PRSB). Notes of all patients admitted to the SAU, over two separate one-week periods, were assessed for the completeness of documentation by two independent doctors. Statistical analysis employed a t-test assuming unequal variance, with a p-value of <0.05 considered significant. The study was considered a service evaluation and was exempt from ethical approval.

Results

During the pre-proforma survey, 100% of respondents felt a proforma would be beneficial, and 77% believed key elements of clerking are missed with the current system. During Cycle 1 (n=62), absence of information was noted in the categories of Responsible Consultant, Medication History, Allergy Status, and Differential Diagnosis. During Cycle 2 (n=119), of 45 assessment criteria, 38 improved (23 significantly; p<0.05), two showed no change, and five were reduced (two significantly; p<0.05). Documentation rates in nine categories improved by over 50%. During the post-proforma survey, 73% of doctors and 86% of allied healthcare professionals (AHPs) agreed that documentation improved with proforma use. Sixty-six percent of clinicians agreed that proformas reduced omission of essential information and provided safe clerking guidance for doctors. One hundred percent of AHPs agreed that the proforma improved handover between shifts.

Conclusion

In a major trauma centre, a standardised proforma improves the completeness of clerking and improves documentation. It also improves the perceived satisfaction of the clinicians and AHPs. The standardised proforma still has a role until the electronic records are implemented.

## Introduction

High-quality medical records are an integral part of Good Medical Practice in the United Kingdom and are an absolute requirement if clinicians are to provide high-quality patient care. In 1965, the Tunbridge report was published; this was the primary work advocating the standardisation of medical records in the United Kingdom. Since this initial publication, there has been an adoption of evidence-based standards for record keeping. This practice has allowed for the identification of deficiencies pertaining to documentation, as well as research-driven strategies to tackle highlighted issues [1].

The importance of improving record keeping is highlighted by the detrimental effects that can arise from poor documentation. There is evidence that poor-quality note keeping is associated with higher rates of adverse events [2] and increased risk of patient death [3]. In addition, accurate, legible, and relevant information within patient notes is crucial during medico-legal proceedings, whereby evidence may be called upon months to years following an event. Litigation proceedings accounted for nearly 2% of the National Health Service (NHS) budget in 2023-2024, equating to £2.87 billion [4]. Ensuring the correct essential information is included in the records can be pivotal to these cases, therefore cementing its importance in litigation. 

Formal guidelines for generic medical record keeping standards have been produced by both the Royal College of Surgeons (RCS) and the Royal College of Physicians (RCP) [5,6], which set out essential information to be included in admission documents. The most recent overarching criteria were outlined in "Standards for the Structure and Content of Health and Care Records", by the Professional Record Standards Body (PRSB) in 2018 [7]. A summary of their recommendations for admission documents is included (see Appendices).

Furthermore, since 2007, the RCP has stated that "data recorded or communicated on admission, handover and discharge should be recorded using a standardised proforma" [8]. The RCS has not adopted this stance formally, despite the NHS Quality and Safety Programme specifying that a "unitary document needs to be in place, issued at the point of entry, which is used by all healthcare professionals and all specialties throughout the emergency pathway" [9], thereby including surgical patients.

A particularly effective method of improving documentation is the use of standardised clerking proformas. Studies show that their implementation produces a number of benefits, without a significant difference in time taken to complete [1]. Documented improvements include enhanced clinician performance [1], greater quality of documented information [10-15], reduced time till review by an experienced clinician [16], and increased audit quality [10,12,17]. Furthermore, a survey conducted by the RCP [18] showed clinicians are "overwhelmingly" in favour of standardised proforma use during admission clerking. Similar preference has been reported by nursing staff [12]. 

Our hospital is a tertiary referral and major trauma centre in the United Kingdom with over 10,000 new surgical patients admitted yearly. The clerking of these patients has historically been carried out by freehand documentation on blank continuation sheets, with no standardisation. The study was designed to assess the quality of surgical admission clerking at a tertiary referral centre, both pre- and post-introduction of a standardised surgical clerking proforma. We used a free publication search engine for key identification factors: "surgical admissions proforma" and "major trauma centre". Since there were no results, we believe this to be the first study of this kind to be undertaken in a major trauma centre. 

## Materials and methods

This was a prospective study conducted in a tertiary referral centre (Derriford Hospital, Plymouth, United Kingdom), with more than 93,000 emergency department attendances per year. The study was conducted in four phases: initial survey of clinicians clerking the patients, audit of surgical clerking documentation, re-audit after the introduction of surgical clerking proforma, and final survey of end users (both clinical and allied healthcare professionals (AHPs)).

Prior to initial data collection, an anonymous survey was designed and distributed to clinicians working within the surgical assessment unit (SAU) via a free-to-use web-based platform, SurveyMonkey® [19], to gather feedback concerning the current clerking system. Both subjective and objective questions were included to rate the quality of the system, as well as to gain an opinion of what clerking doctors would include in a proforma, if one were to be introduced. A proforma was then designed based upon the standards set by both the RCS [5] and the PRSB [7], the survey feedback, and senior consultant guidance. 

Using standards selected from the RCS and PRSB guidance, three assessment sub-categories were identified: "Formal Details", "Clerking Details", and "Management Details". These sub-categories encompassed a total of 45 individual criteria. The "Clerking Details" sub-category was further divided into "Patient History", "Systems Review", and "Patient Examination". A prospective two-cycle audit assessed the quality of clerking data, both before and after the introduction of the proforma. The collection was carried out over two separate one-week periods by two independent doctors, neither of whom was working on the SAU at the time of the study. Notes for all patients admitted to the SAU throughout each week were assessed up to the point of senior review. Emergency trauma, orthopaedic, and elective patient admissions were excluded due to different admission pathways.

Both the first and second cycles were conducted without informing surgical team members (excluding the supervising consultant) to prevent selection bias. The second cycle was undertaken following a two-week period to allow for the familiarisation of clinicians with the new document. Data was collated into a spreadsheet (Microsoft® Excel® for Microsoft 365, Version 2507, Build 19029.20274, Microsoft Corporation, Redmond, Washington, United States) and corroborated with patient list data to avoid duplication and ensure completeness. Statistical analysis was conducted using a t-test, assuming unequal variance, and the pre- and post-implementation data were compared. A p-value of less than 0.05 was considered sufficient to denote statistical significance.

Two months after the introduction, we conducted a further anonymous online user survey (again using SurveyMonkey®) of staff working in the SAU and on surgical wards that receive these patients. Survey respondents included both clinical and AHPs. This determined the impact of the proforma within the SAU, as well as gauging opinion of its efficacy from staff regularly encountering it in their clinical practice on the wards.

The study was registered within the trust's clinical audit department (CA_2018-19-117) for formal approval. The proforma itself was approved by the Health Records Steering Group (HRSG) and registered with the trust's Health Records Documents Management (HRSG 1462/1). The study was considered a service evaluation; hence, formal ethical approval and consent for inclusion in the study were not required. 

## Results

Initial survey response

A total of 13 junior doctors participated in the pre-study questionnaire, with respondents consisting of foundation doctors, core surgical trainees, and specialist registrars. 

Seventy-seven percent of respondents felt that important elements of history and examination of clerking can be missed with the current practice. Subjectively, clinicians felt the following factors often caused issue, or were missed, during clerking: general examination of the respiratory and cardiovascular system can be omitted, when focused abdominal examination takes precedence; social history was often lacking; medication history, particularly allergies; past medical history and past surgical history; accessing relevant previous information that is not electronically recorded; and initial diagnosis and management plan. One hundred percent of the doctors who completed the survey felt that a clerking proforma would be beneficial.

Two-cycle audit results

No notes audited during the period prior to implementation (n=62) were excluded, with only four sets of notes being omitted due to lack of availability following the implementation (n=119). In the first set of data, there were a total of 2790 individual points of data assessed, 2726 (97.7%) of which were available. For the second data set, 100% of the 5175 data points were available.

Out of the 45 assessment criteria, 38 improved, of which 23 were statistically significant. Two out of the 45 showed no change, and five showed a reduction, only two of which were significant (Table 1).

**Table 1:** Comparison of assessed documentation standards pre- and post-proforma implementation Statistical test for significance used: Welch's t-test. * denotes statistically significant results. ECG: electrocardiogram

	Cycle 1	Cycle 2	Difference	P-value	T-value
	Pre-proforma	Post-proforma			
Responsible Consultant Name	22.6%	94.1%	71.5%*	0	-12.387
Assessing Doctor Name	95.2%	95.8%	0.6%	0.848	-0.192
Assessing Doctor Grade	91.9%	94.1%	2.2%	0.596	-0.532
Assessing Doctor Bleep	71%	79.8%	8.9%	0.201	-1.287
Date	100%	91.6%	-8.4%*	0.001	3.29
Time	95.2%	86.6%	-8.6%*	0.041	2.062
Patient Name	100%	100%	0%	-	-
Patient Hospital Number	100%	100%	0%	-	-
Patient Date of Birth	98.4%	100%	1.6%	0.321	-1
Presenting Complaint	95.2%	99.2%	4%	0.168	-1.391
History of Presenting Complaint	95.2%	99.2%	4%	0.168	-1.391
Past Medical History	74.2%	87.4%	13.2%*	0.041	-2.069
Past Surgical Surgery	41.9%	97.5%	55.5%*	0	-8.57
Smoking	37.1%	59.7%	22.6%*	0.004	-2.947
Alcohol	25.8%	52.9%	27.1%*	0	-3.745
Dependence	16.1%	58%	41.9%*	0	-6.396
Medications	45.2%	88.2%	43.1%*	0	-6.129
Allergies	30.6%	87.4%	56.7%*	0	-8.538
Systems Review: Cardiovascular	4.8%	21.8%	17%*	0	-3.624
Systems Review: Respiratory	6.5%	23.5%	17.1%*	0.001	-3.406
Systems Review: Gastroenterological	24.2%	74.8%	50.6%*	0	-7.456
Systems Review: Genitourinary	38.5%	54.6%	16.2%	0.081	-1.77
Systems Review: Gynaecological	2.6%	21%	18.4%*	0	-4.06
Systems Review: Endocrine	0%	2.5%	2.5%	0.618	-0.499
Systems Review: Neurology	0%	16%	16%*	0	-4.735
Systems Review: Musculoskeletal	0%	1.7%	1.7%	0.158	-1.42
Examination	93.5%	95.8%	2.2%	0.539	-0.617
Cardio	14.5%	40.3%	25.8%	0	-4.045
Respiratory	21%	40.3%	19.4%	0.006	-2.808
Abdominal	87.1%	93.3%	6.2%	0.208	-1.268
Vascular	3.2%	17.6%	14.4%	0.001	-3.453
Temperature	29%	84.9%	55.8%	0	-8.356
Respiratory Rate	17.7%	81.5%	63.8%	0	-10.527
Oxygen Saturation	21%	80.7%	59.7%	0	-9.396
Heart Rate	24.2%	84.9%	60.7%	0	-9.483
Blood Pressure	24.2%	83.2%	59%	0	-9.113
Differential Diagnosis	71%	92.4%	21.5%	0.001	-3.407
Management Plan	95.2%	99.2%	4%	0.168	-1.392
ECG	4.8%	11.8%	6.9%	0.089	-1.713
Urine	24.2%	37%	12.8%	0.072	-1.811
Blood	77.4%	86.6%	9.1%	0.144	-1.472
Image	45.2%	69.7%	24.6%	0.002	-3.215
Name	98.2%	93.3%	-4.9%	0.508	0.664
Grade	94.6%	84%	-10.6%	0.081	1.756
Specialty	28.1%	16.8%	-11.3%	0.129	1.531

**Figure 1:**
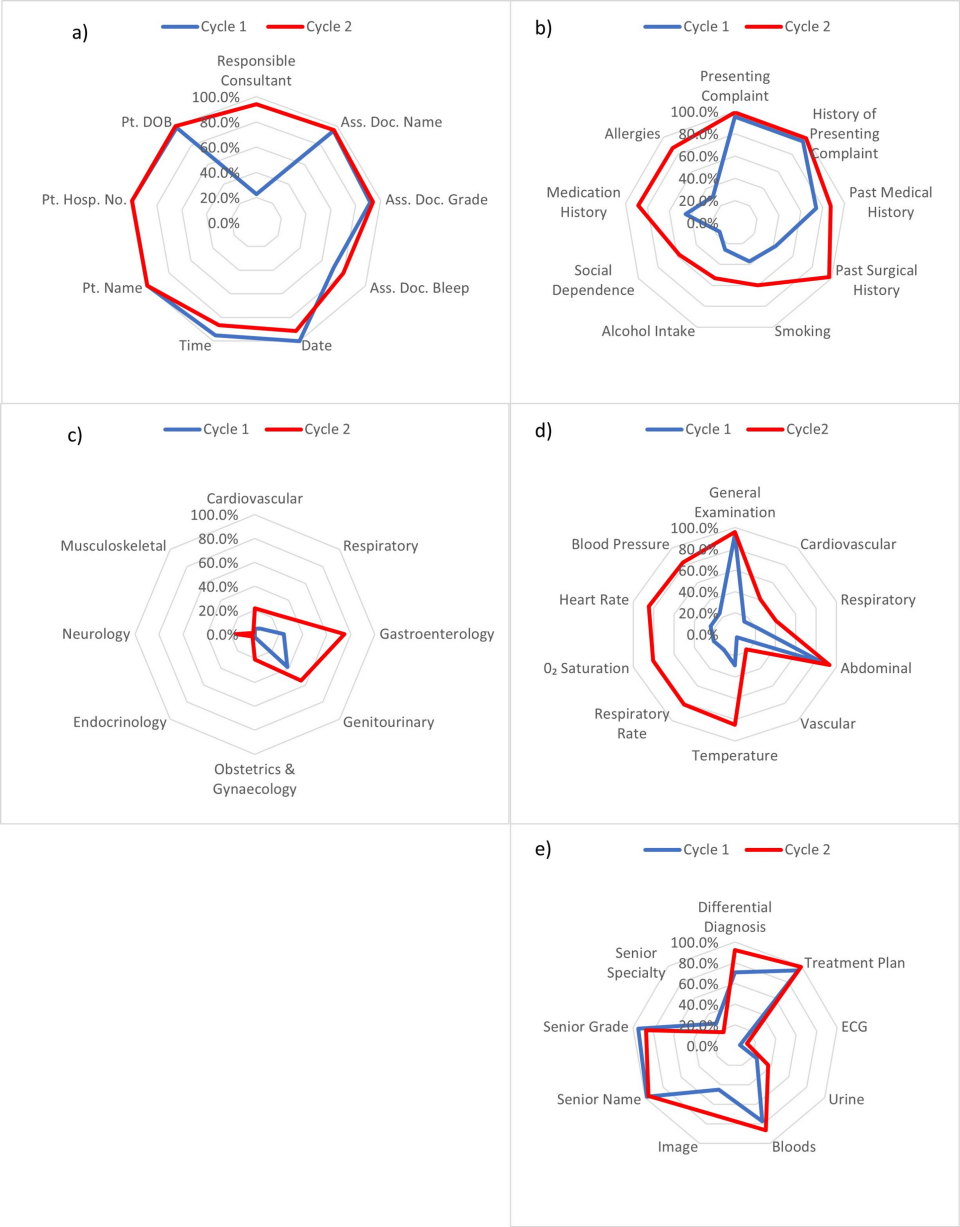
Radar charts demonstrating comparison between audit Cycles 1 and 2 of criteria in (a) "Formal Details", (b) "Clerking Details: Patient History", (c) "Clerking Details: Systems Review", (d) "Clerking Details: Patient Examination", and (e) "Management Details" Pt. DOB: Patient Date of Birth; Pt. Hosp. No.: Patient Hospital Number; Pt. name: Patient Name; Ass. Doc. Name: Assessing Doctor Name; Ass. Doc. Grade: Assessing Doctor Grade;  Ass. Doc. Bleep: Assessing Doctor Bleep Number; ECG: electrocardiogram

Within the "Formal Details" sub-category, eight out of the nine criteria were completed at rates above 80% following proforma implementation (Figure 1). "Assessing Doctor Name", "Assessing Doctor Grade", "Assessing Doctor Bleep", and "Patient Date of Birth" increased by 0.6%, 2.2%, 8.9%, and 1.6% respectively. However, these improvements were not statistically significant. Most notably in this section, there was an increase of over 70% (22.6% before, 94.1% after) in the recording of the responsible consultant (p<0.01). 

When reviewing "Clerking Details" (Figure 1), most components of the "Patient History" improved significantly including "Past Medical History" (p=0.04), "Past Surgical History" (p<0.01), "Smoking" (p<0.01), "Alcohol Intake" (p<0.01), "Social Dependence" (p<0.01), "Medication History" (p<0.01), and "Allergies" (p<0.01). The two remaining criteria, "Presenting Complaint" and "History of Presenting Complaint", had a small improvement of 4%: this was not statistically significant (both 95.2% before, 99.2% after). Further benefits of the proforma were seen in the documentation of "Systems Review", with significant increases in the completion rates of "Cardiovascular" (p<0.01), "Respiratory" (p<0.01), "Gastroenterology" (p<0.01), "Obstetrics and Gynaecology" (p<0.01), and "Neurology" (p<0.01) reviews. "Patient Examination" also showed positive changes, with record of "Cardiac" (p<0.01), "Respiratory" (p<0.01), and "Vascular" (p<0.01) examinations improving significantly, as well as record of "Temperature" (p<0.01), "Respiratory Rate" (p<0.01), "Oxygen Saturation" (p<0.01), "Heart Rate" (p<0.01), and "Blood Pressure" (p<0.01) showing statistically significant increases of over 50%.

A particularly interesting finding is that, although aspects of both "Systems Review" and "Patient Examination" did see considerable improvements, poor documentation rates (under 50%) were still observed, despite proforma introduction. Criteria which this applied to were "Cardiovascular" (4.8% before, 21.8% after), "Respiratory" (6.5% before, 23.5% after), "Obstetrics and Gynaecology" (2.6% before, 21% after), "Endocrinology" (0% before, 2.5% after), "Neurology" (0% before, 16% after), and "Musculoskeletal" (0% before, 1.7% after) reviews and "Cardiovascular" (14.5% before, 40.3% after), "Respiratory" (21% before, 40.3% after), and "Vascular" (3.2% before, 17.6% after) examinations. The only other areas that remained poorly recorded were "ECG" (4.8% before, 11.8% after) and "Urinalysis" (24.2% before, 37% after) results and "Specialty" (28.1% before, 17.4% after) within the senior review. 

In the final area of assessment, "Management Details" (Figure 1), the completeness of documentation of "Differential Diagnosis" (p<0.01) and "Image Findings" (p<0.01) increased significantly. A further four categories were better documented following implementation, "Treatment Plan", "ECG", "Urinalysis", and "Blood" results, although these changes were not significant. There was no significant change seen in the documentation of any aspects of the "Senior Review: Name, Grade, and Specialty". 

Poor recording of data was noted in only two areas of assessment, "Date" (p<0.01) and "Time" (p=0.04); these reduced from 100% to 91.6% and 95.2% to 86.6% before and after the introduction of the structured proforma, respectively. 

**Figure 2:**
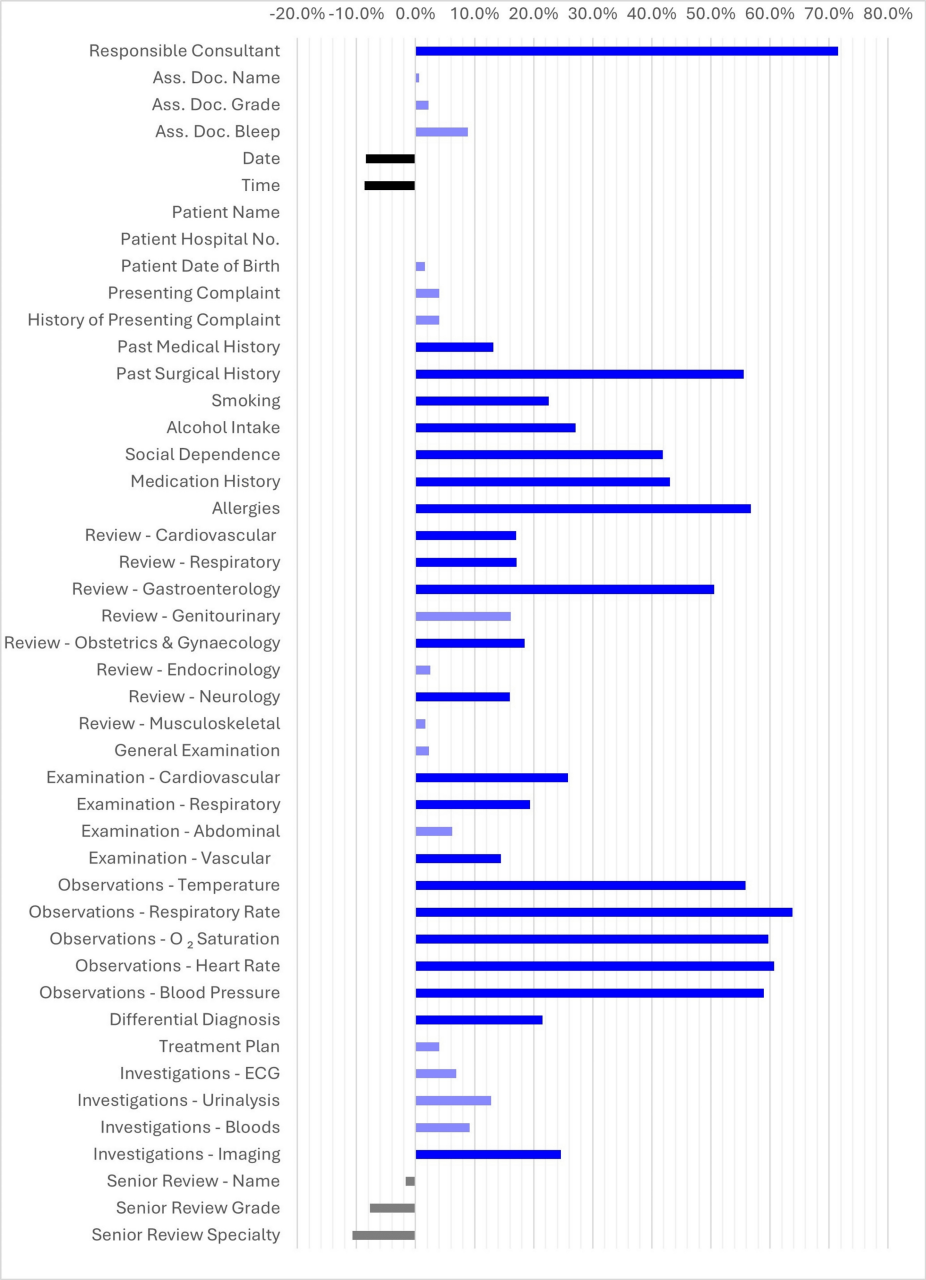
Comparison of the difference in improvements and reductions in documentation Ass. Doc. Name: Assessing Doctor Name; Ass. Doc. Grade: Assessing Doctor Grade;  Ass. Doc. Bleep: Assessing Doctor Bleep Number; Pt. Hosp. No.: Patient Hospital Number; ECG: electrocardiogram

It was noted that before the implementation of the proforma, completion rates were above 80% in 12 criteria, whereas after the proforma was introduced, this occurred in 26 of the criteria. We also noted substantial increases of over 50% in nine categories; these were "Responsible Consultant" (71.5%), "Past Surgical History" (55.5%), "Allergies" (56.7%), "Gastroenterology Systems Review" (50.6%), "Temperature" (55.8%), "Respiratory Rate" (63.8%), "O₂ Saturation" (59.7%), "Heart Rate" (60.7%), and "Blood Pressure" (59%). Figure 2 outlines the comparison of significant and insignificant improvements and deteriorations. There was 100% proforma uptake during the period of observation for this study. 

Post-implementation user survey

Forty-eight responses were received to the follow-up anonymous user survey. Six respondents answered "no" to the initial usage question and therefore did not proceed to the rest of the survey, and five responses were incomplete. This left 37 complete responses: 22 from AHPs (17 registered nurses, two healthcare assistants, and three clerical staff) and 15 from clinicians (eight foundation doctors, three core surgical trainees, and four specialty registrars).

Two-thirds of clinicians agreed, or strongly agreed, that the proforma reduced the chances of essential clinical information being omitted during clerking. The same proportion agreed or strongly agreed that the proforma provided a safe and clear guide for junior doctors during the clerking process. Almost half (47%) disagreed or strongly disagreed that the proforma saved time, with only two agreeing that it did save time.

A notable finding was that 100% of AHPs felt that the handover process improved following the implementation of the clerking proforma. Over 90% (20 staff) agreed or strongly agreed that the proforma's coloured border meant that it was more easily identified in patient notes, compared to freehand clerking sheets.

Further to this, 73% of doctors and 86% of AHPs agreed or strongly agreed that the proforma improved the overall documentation of patient information. Similarly, 84% (31 clinical staff) agreed or strongly agreed that the proforma made accessing relevant patient information easier. 

## Discussion

This study has shown the efficacy of a standardised surgical clerking proforma in improving clerking quality in a major trauma centre, the first study to do so. The basis for high standards of documentation is rooted in the optimisation of patient care and patient safety. Previous work demonstrates that both higher mortality [3] and adverse event [2] rates are associated with poor-quality documentation; prior to our proforma being introduced, 77% of clinicians felt important elements of clerking were missed with freehand documentation. Twenty-three areas of clerking improved significantly following the implementation of a standardised surgical admissions proforma, with 38 out of 45 experiencing some improvement. The majority of clinicians also agreed that the proforma reduced the chance of essential information being omitted. The improvements in clerking quality are not only crucial in achieving the above clinical goals but will importantly confer medico-legal benefits. There is substantial reliance on medical documentation during litigation proceedings, and the agreed stance is that if an event is not documented, then it did not occur [20]. This study has shown that with a proforma, more information is documented during admission. 

Previous studies have demonstrated that proforma use reduces time till senior review [16], improves performance [1], and reduces assessment time [10,21]. Furthermore, they do not take longer to complete [12,22] and actually help clinicians find data, thereby avoiding delays [23]. The benefits these factors can convey continue to be applicable throughout the entire patient journey. Even beyond this, it has been shown that data retrieval for audit is easier [10] and faster [12] with proforma use versus freehand clerking and that audit quality control is more accurate [17]. This contributes to clinical governance and quality improvement, thus improving standards of patient care.

Our data shows that more information is gathered during the initial clerking using a proforma, with the majority of clinicians and AHPs agreeing that access to this information is easier. This will contribute to the more prompt formation of differential diagnoses and initiation of treatments. We have also shown that historic clinical data, such as past surgical history, past medical history, medication history, and allergies, were recorded significantly better following the implementation of the standardised document. With clinicians needing to review notes during a patient's hospital stay, these points being more readily available will mean less time is spent clarifying information from other sources. 

Additionally, with the responsible consultant now being identifiable in over 94% of cases (a significant improvement of over 70%), identifying the team in charge of a patient's care becomes easier. This is particularly relevant in the context of a busy surgical admission unit, with staff working set shift patterns, and patients' clinical conditions evolving independently of these. If the initial clerking doctor is no longer on shift, then other members of the team can be contacted to address any clinical issues that may arise. All these points will contribute to the streamlining of a patient's care pathway and benefit staff productivity.

We should also acknowledge the potential cost-saving role that proforma use has. Where results of certain tests/investigations are not immediately available, it has been documented that repeat ordering occurs [24], leading to a significant cost being incurred over time. Proforma use has been shown to lead to fewer test requests [20], which will help to avoid unnecessary expense and inefficiency. Our study supported this by demonstrating increased documentation of all investigation results, with a significant increase, particularly in the documentation of imaging.

Despite the sizeable improvements in most categories, the completion of certain criteria remained comparably low. This was notable particularly in the social aspects of history, areas of the "Systems Review", and some aspects of examination. Although sometimes these points are overlooked in a surgical clerking, they are vital in assessing a patient's functional status, premorbid state, and cardiorespiratory function, all of which play roles in prognosis, anaesthetic risk, and suitability for surgical intervention.

The importance of education in improving compliance with standards has been highlighted in previous studies [11,17], with Mann and Williams [1] suggesting that it should occur during the induction process and be maintained and reinforced with regular audit and monitoring, which will help in maintaining proper records. We agree that further improvements can be gained with such interventions and that proforma use alone may be insufficient to produce optimal results: targeting clinicians early on in their career will help engender good habits and lead to a career-long impact. With the introduction of interim foundation doctors during the coronavirus pandemic, and the associated alterations to the transition process of final year students to junior doctors that will have to be considered, we have been presented with an opportunity to improve the formal teaching of documentation at an early stage. 

There is a mounting body of research across a broad range of specialties, including paediatrics [25], geriatrics [22], general medicine [10-12,14], and surgery [13,15-17], which support our findings, showing that proforma introduction improves the quality of admission documentation. We feel that the generalisability of the benefits of proforma use, particularly in clerking, is becoming indisputable. The RCP has mandated the use of a proforma during clerking since 2007 [8], whilst the RCS has not yet produced similar guidance. The authors of this study feel that the potential benefit of widespread proforma use warrants such a stance from the RCS, particularly when NHS England (London Region) found that in 2014, only 15 out of 27 emergency surgical departments in acute London hospitals used a standard document issued at admission [9]. There would also be a benefit in reviewing and updating surgery-specific guidelines on medical records and notes, as the most recent ones to be produced date back to 1994 [5]. We hope such guidelines could minimise variation, prevent clinicians from setting their own standards with habitual notation methods, and raise documentation quality nationally. 

Where criteria had been documented well initially, they largely remained so with the introduction of the proforma. We did, however, note two significant instances where documentation had gotten worse: both the date and the time of clerking. We were unable to find a reason for these results. It seems likely that, due to the relative paucity of headings on blank continuation sheets, date and time acted as a structural cue when writing notes on blank forms. With the organisation of the clerking process into a structured document, the typical way in which clinicians completed them may have been slightly disrupted. This issue was minor, with completion rates for date remaining at 91.6% and 86.6%, respectively. As discussed previously, an effective technique to solve this would be the education of clinical staff in comprehensive documentation.

One of the main limitations of this study was that it was not designed to directly measure the impact on patient outcomes or clinical care. Although the findings of this study indicate that proformas improve documentation, we would expect this, combined with the accepted knowledge that poor documentation leads to negative outcomes for patients [2,3], to extend to the improvement of patient care. We also acknowledge that only the presence or absence of data was recorded: no assessment of the content of the recorded data was made. This would have been very difficult to standardise, due to there being limited guidance on the content of surgical clerking, and would have been almost impossible to regulate judgment between observers. We attempted to combat this in our study by agreeing on criteria in advance and discussing individual cases where uncertainties arose. This could also have been addressed with the use of audit tools, such as the "CRABEL" [26] and "STAR" [27] scoring systems, which have been shown to help improve inter-rater reliability. On a wider scale, as mentioned earlier, there is also potential to implement a national consensus to reduce variability between studies. 

A further limitation to the validity of our results is the timeframe over which the study took place and therefore the rotation of staff. With the initial data being taken from one cohort using freehand clerking, and the second set of data being collected after their rotation, we are unable to evaluate independently whether it was purely proforma use or better performance of clinicians that led to improved results. We attempted to limit the impact of this by conducting the study within one academic year to reduce the sampling group, although absolute continuity could not be ensured. Additionally, we attempted to avoid performance bias by not publicising the auditing of the proforma and allowing for a two-week familiarisation period, during which time the data was not audited.

Before we conducted this study, 100% of the survey participants thought that a proforma would be an improvement to the freehand clerking practice that was in place. Following the study, the vast majority of clinicians and AHPs did indeed agree that documentation had improved overall. Over 90% of AHPs felt that the proforma was easier to access, with 100% confirming that it had benefitted the handover process. This is important when considering the sustainability of a study, as approval of clinical staff who regularly use the document is integral to its success and longevity. Similarly, we invited a range of members of the surgical multidisciplinary team to contribute ideas on things they felt should be included in the proforma, thus promoting future compliance. Since carrying out the study, our proforma has been formally adopted by the trust with senior management approval.

Several dates have been set for the elimination of paper medical records, with initial goals dating back to 2008 [1]. When they were not achieved in 2016 [28], a reviewed aim of 2020 was set, and since then, this target has moved further to 2024 [29]. We accept that this means the applicability of this study is limited. However, it has been discussed that standardised documents will be important in working as templates for the creation of computerised versions [1,15,18,25]. With the findings of our study, and several others of this nature, it is in the best interest of the Royal Colleges to work cohesively in the design of computerised proformas. However, care should be taken to avoid such catastrophic results as with previous attempts [30]; to quote Mann and Williams [1], "a mess computerised is just a computerised mess".

## Conclusions

This study has confirmed the role of standardised proforma use in increasing the comprehensiveness of documentation during the clerking of surgical patients, with completion rates of over 80% being achieved in 26 categories. Notably, we saw the biggest improvements in the rudimentary aspects of a surgical clerking, namely, "Consultant Name", "Basic Observations", and "Past Surgical History". This suggests that this improvement can be extended to other specialties and have similar effects on their practices.

## Appendices

**Table 2:** Summary of the Professional Records Standards Body's "Standards for the Structure and Content of Health and Care Records" NHS: National Health Service

Subheading	Element
Patient demographics	Name
	Date of birth
	NHS number (or other identifier)
Social context	Household composition
	Occupational history
	Smoking status
	Alcohol status
	Services and care
Admission details	Date/time of admission
	Responsible consultant
	Specialty
Problems and issues	Examination findings, symptoms, and signs, which are likely to have relevance and are not a diagnosis
History	Presenting complaint
	History of presenting complaint
	Past medical, surgical, or mental health history
Family history	
Medications	
Investigation results	
Allergies	
Examination findings	
Clinical review of systems	
Plan and requested action	
Person completing record	Name
	Role
	Grade
	Specialty
